# *Ex vivo* diffusion MRI of the human brain: Technical challenges and recent advances

**DOI:** 10.1002/nbm.3941

**Published:** 2018-06-04

**Authors:** Alard Roebroeck, Karla L. Miller, Manisha Aggarwal

**Affiliations:** ^1^ Department of Cognitive Neuroscience, Faculty of Psychology & Neuroscience Maastricht University Maastricht the Netherlands; ^2^ FMRIB Centre University of Oxford Oxford UK; ^3^ Department of Radiology Johns Hopkins University School of Medicine Baltimore MD USA

**Keywords:** cortical layers, diffusion MRI, *ex vivo*, gray matter, validation, white matter

## Abstract

This review discusses *ex vivo* diffusion magnetic resonance imaging (dMRI) as an important research tool for neuroanatomical investigations and the validation of *in vivo* dMRI techniques, with a focus on the human brain. We review the challenges posed by the properties of post‐mortem tissue, and discuss state‐of‐the‐art tissue preparation methods and recent advances in pulse sequences and acquisition techniques to tackle these. We then review recent *ex vivo* dMRI studies of the human brain, highlighting the validation of white matter orientation estimates and the atlasing and mapping of large subcortical structures. We also give particular emphasis to the delineation of layered gray matter structure with *ex vivo* dMRI, as this application illustrates the strength of its mesoscale resolution over large fields of view. We end with a discussion and outlook on future and potential directions of the field.

**Abbreviations used**
2D/3D: two−/three‐dimensional
ADC: apparent diffusion coefficient
ADD: axonal diameter distribution
CPMG: Carr–Purcell–Meiboom–Gill
CSD: constrained spherical deconvolution
DBS: deep brain stimulation
DEC: direction‐encoded color
dMRI: diffusion magnetic resonance imaging
DRTT: dentatorubrothalamic tract
DSI: diffusion spectrum imaging
DTI: diffusion tensor imaging
DW: diffusion‐weighted
DW‐RARE: diffusion‐weighted multi‐shot 3D rapid acquisition with relaxation enhancement
dwSE: diffusion‐weighted spin‐echo
dwSSFP: diffusion‐weighted steady‐state free precession
dwSTE: diffusion‐weighted stimulated echo
EM: electron microscopy
EPI: echo planar imaging
FA: fractional anisotropy
FOD: fiber orientation distribution
GM: gray matter
GRASE: gradient and spin‐echo
HARDI: high angular resolution diffusion imaging
HCP: human connectome project
LM: light microscopy
NMR: nuclear magnetic resonance
OCT: optical coherence tomography
PAS: persistent angular structure
PBS: phosphate‐buffered saline
PLI: polarized light imaging
PMI: post‐mortem interval
QBI: *q*‐ball imaging
RARE: rapid acquisition with relaxation enhancement
RF: radiofrequency
ROC: receiver operating characteristic
SI: scan interval
SNR: signal‐to‐noise ratio
SOCS: serial optical coherence scanner
SS‐EPI: single‐shot echo planar imaging
STA: structure tensor analysis
STEAM: stimulated echo acquisition mode
TE: echo time
TR: repetition time
UHF: ultrahigh field
WM: white matter

## 1 INTRODUCTION

**Figure 1:**
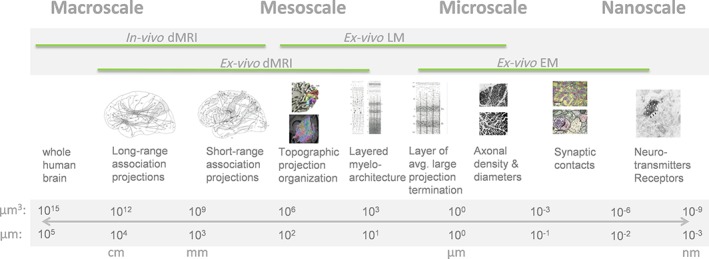
The multiscale nature of human structural brain connectivity and its measurement with different techniques. The measurement of the connectivity phenomena here refers to features directly resolved by the acquired spatial resolution of the technique (not by modeling the contrast over multiple measurements and indirectly inferring statistics of such features, as in microstructure modeling of diffusion MRI). dMRI, diffusion MRI; EM, electron microscopy; LM, light microscopy

Diffusion magnetic resonance imaging (dMRI) can be used to non‐invasively probe the connectivity and microstructure of human brain tissue *in vivo*. At diffusion times of the order of a few tens of milliseconds, the diffusion‐weighted (DW) MR signal is sensitive to micrometer‐scale tissue properties averaged over the imaging voxel size, e.g. intracellular and extracellular volumes and the orientations of sub‐voxel microscopic structures. The typical voxel size for *in vivo* dMRI is in the range of 1–3 mm, whereas, for *ex vivo* dMRI (i.e. dMRI of excised tissue, either *post mortem* or from resection surgery), it can reach the level of a few hundred micrometers. To highlight the role of *ex vivo* dMRI in human connectomics and the investigation of neuroanatomy, it is interesting to consider the many orders of magnitude over which brain connectivity is organized in three‐dimensional (3D) space. Figure 1 illustrates the multi‐scale nature of structural connectivity in the human brain, and available imaging modalities to probe connectivity at each scale. This continuum of connectivity scales can be usefully divided into the macroscopic, mesoscopic, microscopic and nanoscopic scale (or macroscale, mesoscale, microscale and nanoscale) without sharply defined borders. Each imaging modality (*in vivo* and *ex vivo* dMRI, light microscopy and electron microscopy) is specific to the mapping of the connectivity over a single spatial scale or two scales at most, reflecting its typical resolution and field of view. This, in turn, means that each modality is well suited to the characterization of specific tissue features of interest, such as long‐range association projections, layered intracortical myeloarchitecture or synaptic contacts.

*In vivo* dMRI has played an important role in the subfield of human macroscale connectomics, spearheaded in recent years by the Human Connectome Project,^1^, ^2^ in which macroscale long‐range and short‐range association projections have been mapped with *in vivo* dMRI and tractography, together with functional MRI measurements and behavioral and genetic measures. At the other end of the continuum, light microscopy (LM) and electron microscopy (EM) measurements have dominated microscale and nanoscale connectomics studies, aimed at single‐ or few‐axon trajectories and synaptic contacts, performed almost exclusively in small rodents.^3^, ^4^, ^5^, ^6^, ^7^, ^8^

The role of *ex vivo* dMRI in this continuum of structural connectivity scales is two‐fold. First, the combined potential to achieve higher spatial resolution than with *in vivo* dMRI and larger 3D fields of view than with LM makes it ideal for the mapping of mesoscale connectivity, such as subcortical projection systems and layered intracortical projections. Second, with the introduction of sophisticated *q*‐space sampling schemes, advanced tractography and fiber orientation distribution (FOD) reconstruction methods, there is an increasing need to validate these techniques in the human brain, which cannot be performed *in vivo*. In this respect, the overlap of *ex vivo* dMRI with LM in terms of spatial resolution in the mesoscopic scale and the possibility of performing both dMRI and LM on the same tissue are crucial to enable the evaluation of different diffusion processing techniques and to reliably compare diffusion modeling approaches with a robust ground truth measurement. However, the acquisition of high‐quality *ex vivo* dMRI measurements faces considerable challenges. The largest of these stem from the altered properties of fixed post‐mortem tissue compared with the *in vivo* case, which are mostly detrimental to the quality of dMRI measurements. This means that standard *in vivo* dMRI acquisition techniques are typically unsuitable and, instead, *ex vivo* dMRI relies on highly specialized acquisition strategies.

In this review, we discuss both cutting‐edge *ex vivo* dMRI techniques and summarize recent *ex vivo* dMRI validation studies and neuroanatomy investigations, with a focus on the human brain. In Section 2, we review the challenges posed by the properties of post‐mortem tissue, and the tissue preparation and pulse sequence techniques available to tackle these, especially for large samples or intact brains. In Section 3, we review *ex vivo* dMRI studies of the human brain, highlighting the validation of white matter (WM) orientation estimates and the atlasing and mapping of large subcortical structures. We also give particular emphasis to the delineation of layered gray matter (GM) structure with *ex vivo* dMRI. In Section 4, we end with a discussion of open issues and an outlook on where the field might be moving next.

## 2 *EX VIVO* DIFFUSION ACQUISITION

The main challenges for high‐resolution *ex vivo* human dMRI studies are the strongly reduced *T*_2_ and diffusivity of fixed tissue. The main tools in the hands of the investigator to combat these include high gradient strength (mostly in the range 140–1500 mT/m for preclinical systems), longer scanning times, close‐fitting radiofrequency (RF) coils and optimized MR pulse sequences. The use of small‐bore preclinical magnet systems can afford higher spatial resolutions and signal‐to‐noise ratio (SNR) than typically achievable with current clinical hardware, but will also limit sample size. In this section, we first focus on human *ex vivo* sample properties and preparation, and then continue with MR pulse sequences tailored for *ex vivo* diffusion MR acquisitions.

### 2.1 Sample properties and preparation

Formalin‐ or paraformaldehyde‐fixed human brain tissue has reduced proton density, *T*_1_, *T*_2_ and overall diffusivity compared with living subjects, because of the effects of death, tissue fixation and lower than body temperature. The most important hindrances to the acquisition of high‐quality *ex vivo* human dMRI data are the reduced *T*_2_ and diffusivity of fixed tissue, which strongly reduce the contrast‐to‐noise ratio in dMRI. This is because low *T*_2_ causes the spin‐echo signal to decay quickly, whereas low diffusivity leads to a need for stronger diffusion weighting (longer diffusion times and higher *q* and *b* values) to probe similar length scales, which increases the echo time (TE) at which the signal can be acquired.

An important difference between human *ex vivo* studies and animal *ex vivo* studies is the way in which brain tissue is preserved and fixed. In most animal studies, the post‐mortem interval (PMI; the elapsed time between death and tissue fixation) can be very short, by following sacrifice of the animal immediately with fixation procedures. Very often perfusion fixation by pre‐mortem intracardial injection of fixative is performed (effectively achieving a PMI of zero). In contrast, in human *ex vivo* studies, the PMI is always longer and perfusion fixation can only be performed *post mortem* by mechanical pumping of fixative through the cadaver, for instance through the femoral artery. Perfusion fixation is usually followed by post‐fixation through immersion in fixative. In the large human brain, immersion fixation will be less effective than in smaller animal brains, simply because of the long times necessary for passive diffusion of fixative over large distances.^9^ In many cases, mechanical perfusion fixation is not available or undesired (because of the risk of microscopic damage induced by the pressure of pumping) and human brain tissue is fixed through immersion only. The effects of longer PMI and less effective perfusion and immersion fixation generally cause the quality of fixed human tissue to be lower than that of animal tissue.

As a reference, in animal experiments based on immersion fixation (rather than intracardial perfusion fixation), considerable *T*_1_, *T*_2_ and diffusivity reductions have already been reported in post‐mortem brain tissue. For instance, Shepherd et al.^10^ reported a 21% reduction in *T*_1_ and 81% reduction in *T*_2_ for rat cortical slices immersion fixed in 4% paraformaldehyde compared with the fresh unfixed post‐mortem values. This indicates that a large part of the reduction in *T*_2_ is caused by fixation alone, probably through a combination of dehydration and unbound fixative. Indeed, prolonged washing in saline or phosphate‐buffered saline (PBS) to wash out fixative and rehydrate the sample has been reported in this study to fully restore reductions in *T*_2_ to fresh unfixed post‐mortem values (but not *in vivo* values).^10^ As *T*_1_ is dominated by the size of molecules in the tissue environment, the *T*_1_ reduction is probably caused by the cross‐linking of proteins, which would make this effect fundamental to fixed tissue. In a study of primate brains, apparent diffusion coefficients (ADCs, which we denote as *D*), measured with dMRI, have been reported to be reduced by as much as 50% in unfixed *ex vivo* WM and 80% in fixed WM with respect to *in vivo* values.^11^ Diffusion is reduced partly because of the lower (room) temperature at which post‐mortem sample acquisitions are typically performed and partly because of the effects of formalin fixation and dehydration. Again, washing fixed tissue in saline can counter some of these effects and has been reported to raise WM ADC by approximately 30%.^11^

In human *ex vivo* tissue, with its less ideal fixation conditions, reductions in *T*_1_, *T*_2_ and diffusivity have similar magnitudes. Pfefferbaum et al.^12^ reported a *T*_1_/*T*_2_ reduction from *in vivo* to fixed tissue of 63%/35% for WM and 76%/33% for cortical GM, using a 1.5‐T clinical MRI system. McNab et al.^13^ reported human WM *T*_1_/*T*_2_ values of 340 ms/45 ms (similar to Miller et al.^14^) at 3 T, which would amount to reductions of approximately 60%/35%. Foxley et al.^15^ reported human WM *T*_1_/*T*_2_ values of 520 ms/55 ms at 3 T and 595 ms/37 ms at 7 T, which would amount to reductions of approximately 40%/29% at 3 T and 50%/33% at 7 T with respect to *in vivo* values. Both of the last two studies reported a WM diffusivity of 0.8 × 10^−4^ mm^2^/s, which corresponds to an approximate 85% reduction compared with *in vivo* values, with a GM diffusivity of two to three times higher than that of WM *ex vivo*, which is comparable with the relative WM/GM value *in vivo*. It should be noted that all of these values were measured in intact fixed human brains which were not washed in saline and were obtained with different fixation methods and PMIs between studies. Furthermore, *T*_1_ and *T*_2_ values are dependent on the field strength, whereas *D* estimated by a monoexponential fit is dependent on the *b* value (different in these studies), affecting the interpretation of these reductions.

Beyond an overall decrease in mean diffusivity, effects of fixation are more subtle. Diffusion anisotropy, measured with the fractional anisotropy (FA), has been reported to remain largely intact in animal studies.^16^, ^17^ Richardson et al.^18^ performed a controlled comparison in rat optic nerve tissue, comparing viable isolated tissue and fixed tissue in identical conditions at physiological temperature, and demonstrated lower axial diffusivity and FA and higher radial diffusivity, pointing to an altered microstructural environment. Most human *ex vivo* studies have reported reductions in FA in fixed tissue of 50–60% in both WM and GM.^13^, ^19^ Overall, fixation maintains the orientation of anisotropy and seems to preserve the broad microstructural environment of brain tissue, but could change aspects of the microenvironment relevant to dMRI, such as increased membrane permeability and extracellular volume.^10^, ^20^

The effect of PMI and scan interval (SI, time from death to scan) on the quality of human *ex vivo* tissue is also of concern, especially considering the large sample size and use of immersion fixation where the fixative reaches deep tissue only after a considerable time.^21^, ^22^ Animal studies have demonstrated considerable effects of PMI on FA and ADC,^21^ but have also suggested that, after fixation, tissue is stable for SI of up to 3 years.^23^ Shepherd et al.^20^ performed a controlled rat spinal cord tissue study comparing perfusion fixation with increasing PMIs for immersion fixation. Diffusivity decreased by 20–40% in the first 6 h of PMI for both spinal WM and GM and by another 20–40% from 6 h to 24 h. They observed a 38% decrease in FA of WM in the first 6 h and a 52% decrease in FA by 24 h PMI. In a human *ex vivo* study with long and variable PMIs and SIs, the considerable influence of PMI was corroborated.^19^ Multiple regression analysis of PMI and SI indicated that PMI is the primary driving factor in reducing diffusivity, with *D* reduced by (0.1–0.2) × 10^−4^ mm^2^/s per additional hour PMI. The dependence of diffusion parameters on PMI suggests that degradation, for example from autolysis in the period prior to fixation, is a major cause of the differences between *ex vivo* and *in vivo* tissue. Taken together, this suggests that *ex vivo* tissue quality, as measured by its diffusivity, deteriorates with increasing PMI. The effect of SI on diffusivity is much more limited, implying that high‐quality, well‐fixed samples can be used for *ex vivo* dMRI studies for a considerable time, up to several years.

For the above reasons, specimens with a low PMI should be preferred whenever possible, but even very low PMI samples must contend with strongly reduced *T*_2_ and diffusivity. The adverse effects of lower *T*_2_ and diffusivity are partly compensated by reduced *T*_1_ for *ex vivo* tissue, allowing more SNR efficiency at short TRs. This can be further taken advantage of by soaking small specimens in gadolinium‐based contrast agents and reducing *T*_1_ even further.^11^ Techniques available to counter the loss in water diffusivity, and therefore to (partly) avoid raising *b* values at the cost of SNR, include increasing the sample temperature during scanning (e.g. Bastiani et al.,^24^ where this was achieved with an animal warming system for a small sample), which raises WM diffusivity by approximately 95% for a 20°C increase,^11^ in line with a measured 64% increase in *D* from 15 to 35°C.^25^ However, the sensitivity of diffusivity to temperature also requires tight monitoring and control of a steady temperature, as temperature fluctuations could induce considerable uncontrolled signal fluctuations. The requirement for a heating system that can achieve a considerable controlled temperature increase over a long time, together with a concomitant increase in thermal noise in RF coil electronics with increasing temperatures, has precluded a temperature increase from becoming a commonly applied technique. For similar reasons, Dyrby et al.^23^ recommended allowing temperature and unstable tissue mechanics to stabilize prior to actual data acquisition. As noted above, an effective and therefore common approach to improve the diffusion signal is prolonged washing in saline or PBS to wash out fixative and rehydrate the sample, which partly restores reductions in both *D* and *T*_2_. However, similar to initial immersion fixation, PBS washing of very large tissue samples, such as intact human brains, can be problematic because of the limited passive penetration of the buffer solution to the center of the tissue.^19^, ^21^ It is therefore not commonly applied for intact human brains. Finally, a common approach to improve *ex vivo* dMRI scanning conditions is to embed the sample in a proton‐free (and therefore ^1^H MRI‐invisible) and tissue susceptibility‐matched fluid, such as Fomblin or Fluorinert. This avoids high signal from water‐based embedding fluid causing clipping or ringing signal in low *b* (or *b*_0_) volumes, and simultaneously avoids off‐resonance distortions at the tissue edges because of the matched magnetic susceptibility.

### 2.2 Pulse sequences for *ex vivo* dMRI

The changes in post‐mortem tissue properties discussed above render the application of protocols routinely used for *in vivo* diffusion imaging (e.g. pulsed‐gradient spin‐echo sequences with single‐shot echo planar imaging readout) impractical for most *ex vivo* diffusion experiments. This is true for both the diffusion weighting preparation and the imaging readout phases of the diffusion pulse sequence, which we discuss sequentially.

#### 2.2.1 Diffusion weighting preparation

**Figure 2:**
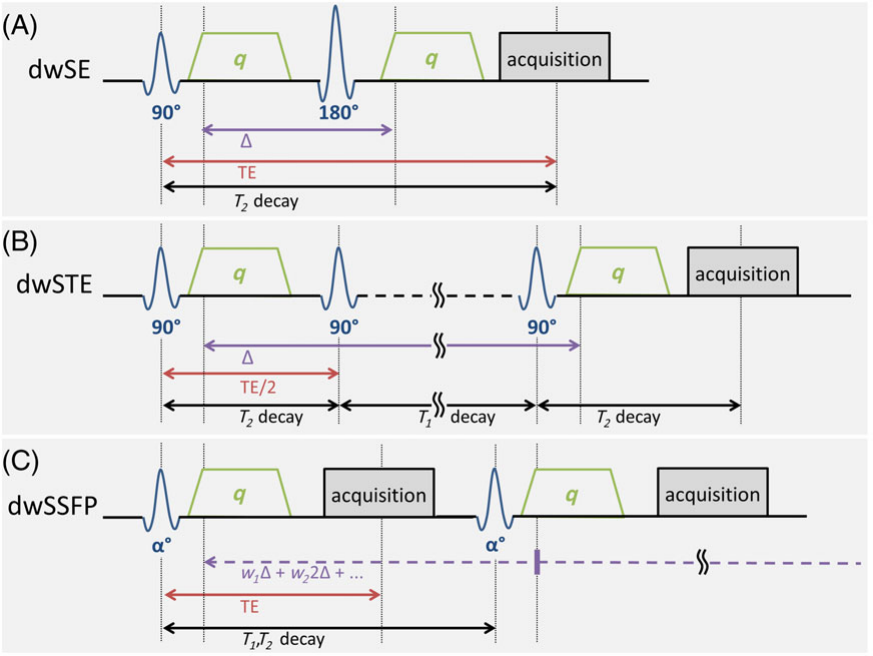
Diffusion‐weighted radiofrequency (RF) pulse sequences. (A) Diffusion‐weighted spin echo (dwSE) or pulsed‐gradient spin‐echo. (B) Diffusion‐weighted stimulated echo acquisition mode (dwSTE). (C) Diffusion‐weighted steady‐state free precession (dwSSFP). For each sequence, the echo time (TE), diffusion time (Δ) and periods over which *T*_1_, *T*_2_ or combined *T*_1_ and *T*_2_ decay occurs are indicated

Diffusion weighting typically uses a spin‐echo (diffusion‐weighted spin‐echo (dwSE); Figure 2A) pulse sequence with two identical, large gradient pulses before and after the refocusing pulse.^26^ The effect of these gradients is quantified by the ‘*b* value’, which is proportional to the product of *q* (quantifying the gradient pulse moment or area) squared and Δ (quantifying the gradient pulse spacing). The image contrast for tissue with diffusion coefficient *D* is proportional to exp(−*bD*), and a common target contrast is to achieve *b* = *D* ^−1^.^27^ In order to achieve a given target contrast, one therefore wishes to maintain the product *bD*; this can be problematic in fixed post‐mortem tissue because of the reduction in both *D* and *T*_2_ discussed above. If we wish to achieve comparable contrast to *in vivo* experiments, the reduction in *D* dictates a higher *b* value, which, in the face of limited maximum gradient strength, directly dictates longer gradient pulses; unfortunately, these longer pulses then require longer TEs, which results in extremely low signal levels because of reduced *T*_2_. Post‐mortem imaging of small animal brain samples on preclinical systems with high gradient strength were able to achieve excellent data quality with spin‐echo sequences using volumetric excitations, short repetition time (TR) (due to reduced *T*_1_) and 3D *k*‐space encoding.^11^ Unfortunately, large tissue samples or whole human brains can only be accommodated in clinical scanners with more limited gradient strength, under which circumstances this approach suffers from low signal.^19^

An alternative to spin‐echo‐based diffusion preparations is stimulated echoes [diffusion‐weighted stimulated echo (dwSTE)^28^], in which the spin‐echo refocusing pulse is replaced with two 90° pulses (Figure 2B). In the time interval between these pulses, the magnetization is stored along the longitudinal axis, where it decays more slowly according to the long *T*_1_ relaxation time, rather than *T*_2_. This can be used to achieve long diffusion times, which increases diffusion contrast as molecules explore their local environment more fully.^29^ Unfortunately, stimulated echo sequences incur a signal loss overhead of a factor of 2, which corresponds to the amount of magnetization that is not stored longitudinally. In practice, this overhead has limited the attraction of stimulated echo sequences unless particularly long diffusion times are desired.^30^ One potential sequence to overcome this limitation is the hyper‐echo,^31^ which is able to refocus this lost signal^32^; however, this route remains relatively unexplored.

Another alternative is the diffusion‐weighted steady‐state free precession (dwSSFP; Figure 2C) sequence.^33^, ^34^ SSFP is able to retain signal over multiple TR periods,^35^ essentially acquiring a plurality of spin and stimulated echoes simultaneously. The result is both strong diffusion weighting and high SNR, even for short *T*_2_ tissues.^14^ In addition, this means that the dwSSFP signal is a much more complicated function of flip angle, *T*_1_, *T*_2_ and *b* value,^35^ requiring an alternative signal model.^36^ Importantly, one cannot assign a single *b* value or diffusion time to SSFP, as the signal results from a combination of spin‐echo and stimulated echo contributions, such that the diffusion weighting accumulates over multiple diffusion times. The use of dwSSFP *in vivo* has, to date, been essentially precluded because of its motion sensitivity, but it was one of the first methods used to acquire high‐quality diffusion imaging in whole human brains.^13^

The considerations above mean that the discussed pulse sequences behave differently under different field strengths, particularly when moving from 3 T to ultrahigh fields (UHFs; 7 T and above). For dwSE to remain effective for *ex vivo* tissue at UHF, TEs would have to become prohibitively short, as *T*_2_ decreases with increasing field strength. For dwSTE and dwSSFP, the move to UHF is more beneficial, providing more SNR/unit time than 3 T. This is because *T*_2_ (which dominates dwSE) decreases, but *T*_1_ increases, which is leveraged in SSFP and stimulated echo acquisition mode (STEAM). In a comparison study, dwSSFP was found to have considerably higher SNR/unit time at 7 T compared with 3 T and to achieve excellent quality diffusion imaging at 7 T in post‐mortem, whole human brains.^15^ Using dwSTE at 9.4 T, high‐quality whole *ex vivo* human brain dMRI was reported at 400 μm isotropic.^37^

#### 2.2.2 Imaging readout and echo trains

Single‐shot echo planar imaging (SS‐EPI) is the widely used readout of choice for *in vivo* human brain dMRI, and offers the advantage of speed and high efficiency. However, SS‐EPI readout is suboptimal for *ex vivo* dMRI for two reasons. First, the lower *T*_2_ and lower proton density of fixed tissue^12^ pose practical constraints on the maximum allowable TE for *ex vivo* dMRI. This precludes the use of long TEs typically required for SS‐EPI readouts, which traverse all of *k*‐space following a single diffusion preparation period. Short *T*_2_ also leads to blurring because of signal decay during long SS‐EPI readouts, making these readouts inappropriate for achieving high spatial resolution. In addition, the higher *b* values required compared with *in vivo* experiments are often accompanied by stronger eddy currents induced by the resulting strong diffusion‐sensitizing gradients. These are particularly problematic when combined with long SS‐EPI readouts, leading to profound geometric distortion artifacts.

Multi‐shot or segmented EPI, which combines interleaved *k*‐space trajectories from multiple excitations, offers one approach to mitigate the SNR loss associated with SS‐EPI *k*‐space traversal. Recent studies have demonstrated 3D segmented EPI‐based dMRI of the whole post‐mortem human brain at submillimeter isotropic resolutions on human (3 T) scanners,^19^, ^38^ and high‐resolution (250 μm isotropic) dMRI of the intact human brainstem at high field (11.7 T).^39^ Segmented EPI has the advantage of being highly efficient, and is achievable through the modification of spin‐echo EPI sequences used for *in vivo* diffusion imaging.^19^ One intrinsic drawback of EPI, however, is its high sensitivity to inhomogeneous *B*_0_ fields, which leads to geometric distortions in areas of susceptibility changes (e.g. air–tissue interfaces) and in the presence of gradient‐induced system eddy currents – problems that are only partially mitigated with segmented EPI and exacerbated with increasing field strength.

dwSE sequences with single‐line readouts have also been used for dMRI of the *ex vivo* brain,^23^, ^40^, ^41^ but are generally limited to two‐dimensional (2D) multi‐slice acquisitions because of the reduced scan efficiency η (= SNR/√scan time). 3D *k*‐space trajectories employing echo trains with multiple refocusing RF pulses offer increased resolution and SNR over EPI, with less geometric distortions, albeit at the expense of speed and higher sensitivity to transmit RF (*B*_1_^+^) inhomogeneities. These include DW multi‐shot 3D rapid acquisition with relaxation enhancement (DW‐RARE) sequences,^42^ where multiple spin echoes are acquired per shot with a series of 180° refocusing pulses. DW 3D gradient and spin‐echo (DW‐GRASE) acquisition, which interleaves RF refocusing pulses with trains of alternating polarity readout gradient lobes, offers increased efficiency compared with RARE and higher SNR with less geometric distortion than EPI.^43^, ^44^ Accelerated *k*‐space traversal with 3D‐GRASE readout has been used for high spatial and/or angular resolution dMRI of fixed brain tissue on UHF systems with minimal distortion.^45^, ^46^

**Figure 3:**
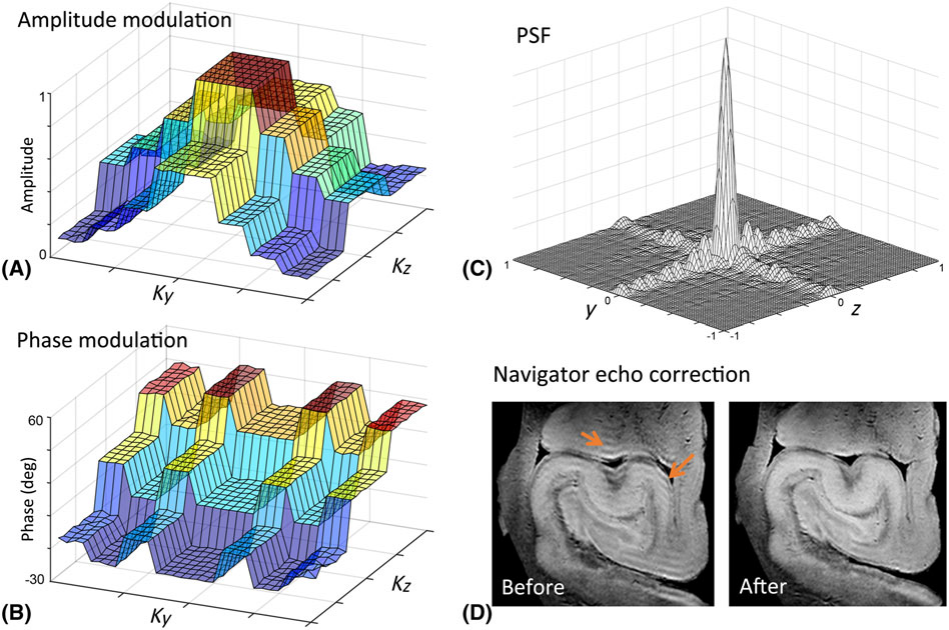
An example showing amplitude and phase modulations in *k*‐space and navigator echo‐based phase correction for three‐dimensional (3D) diffusion‐weighted gradient and spin echo (GRASE) readout. (A) *T*_2_‐ and *T*_2_*‐dependent amplitude modulation along *K*_*y*_ and *K*_*z*_ for ‘center‐out’ *k*‐space sampling with echo planar imaging (EPI) factor = 3 and rapid acquisition with relaxation enhancement (RARE) factor = 4. (B) Corresponding phase modulation in *k* space as a result of off‐resonance spins and phase oscillations between odd‐ and even‐numbered echoes. (C) Resulting point spread function (PSF). (D) Diffusion‐weighted image from a post‐mortem human hippocampus before and after phase correction. Phase discontinuities in *k* space result in ghosting artifacts (arrows) in diffusion‐weighted images, which are minimized by twin‐navigator echo phase correction. (Aggarwal et al.^43^)

The use of refocusing pulse trains in DW pulse sequences, particularly at higher field strengths, requires sophisticated methods to correct for signal phase errors. If uncorrected for, these errors lead to severe ghosting and blurring artifacts in the reconstructed DW images acquired using multi‐RF‐pulse readouts (as illustrated in Figure 3). The strong diffusion‐weighting gradients necessary for *ex vivo* dMRI introduce incoherent phase shifts that cause the Carr–Purcell–Meiboom–Gill (CPMG) condition to be violated, and also result in unstable phase oscillations between echoes acquired after odd‐ and even‐numbered RF pulses.^43^, ^47^ Phase cycling and the use of crusher gradients around the refocusing RF pulses are therefore necessary to suppress unwanted coherence pathways. In DW‐GRASE, phase accrual as a result of off‐resonance effects and phase oscillation between odd and even spin echoes can be separated along different phase‐encoding axes by optimizing the 3D *k*‐space trajectory (Figure 3). This enables the use of a twin‐navigator echo correction scheme^43^, ^48^ to minimize ghosting artifacts arising from phase modulation across *k*‐space. As illustrated in Figure 3D, the twin‐navigator echo correction can minimize the ghosting and blurring artifacts in the resulting DW images. The use of reference scans has also been proposed to correct for phase errors arising from residual eddy current effects.^42^

It is important, at least conceptually, to separate phase errors and resulting artifacts within diffusion volumes from signal drift across different volumes, mostly caused by drift away from the resonance frequency. Signal drift can be corrected by online hardware frequency feedback or by acquiring interspersed non‐diffusion‐weighted (*b*_0_) images over the scan duration, from which the decrease in signal magnitude over time can be estimated and compensated. This is similar to *in vivo* dMRI studies (e.g. Vos et al.^49^) and is not specific to post‐mortem dMRI. More generally, pre‐ and post‐processing of *ex vivo* dMRI data overlap with the *in vivo* case to a great extent, except in analysis which depends on relaxation parameters (which need to be adjusted to the lower *ex vivo* values) and *post‐hoc* motion and distortion correction (which can often use simpler affine transformation correction methods *ex vivo*).

## 3 NEUROANATOMICAL STUDIES AND VALIDATION WITH *EX VIVO* dMRI

A large number of investigations in recent years have used the techniques described above to examine human brain tissue samples with dMRI. Although the purposes of these investigations are diverse, most include at least one of the two objectives discussed at the start of this review: validation and mapping of mesoscale connectivity. In Section 3.1, we focus on the validation of model fitting and inference techniques, in particular those concerning diffusion orientation and tractography. Subsequently, we discuss neuroscience investigations into the architecture of brain tissue at mesoscopic scales. We separate this application into atlasing and mapping subcortical structures, discussed in Section 3.2, and detection of cortical lamination, discussed in Section 3.3.

### 3.1 Validation of orientation estimates and tractography

**Figure 4:**
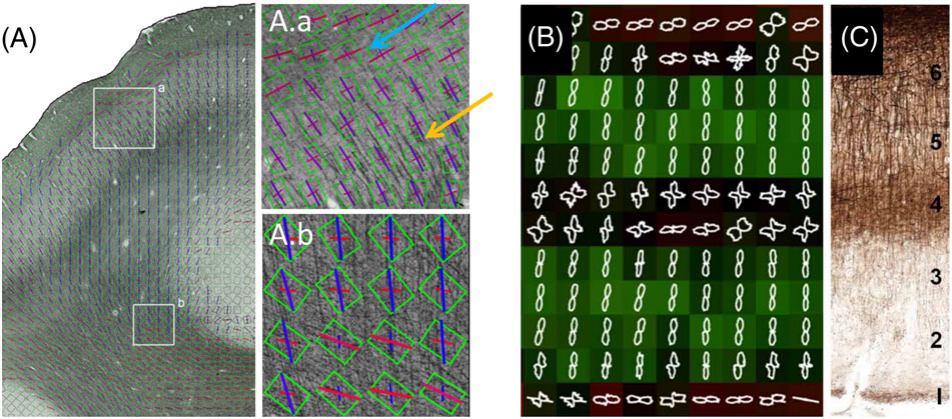
Validation of orientation estimates in human cortex. (A) Gray matter (A.a) and white matter (A.b) locations with multiple fiber orientations estimated with structure tensor analysis (STA) of a myelin stain (orientation color coded between red = horizontal and blue = vertical) and the diffusion tensor (green box: axes of largest diffusion projected into the section plane).^58^ In (A.a), the orientation of the main tensor axes (green box) is well aligned with the main radial STA (purple) in the deeper layers (orange arrow), but tensor orientation accuracy is less in cortical layers with both radial and tangential intracortical fibers (blue arrow). In (A.b), the orientation of the main tensor axes (green box) is misaligned with the STA orientations (red and blue). Two‐dimensional (2D) fiber orientation distributions (FODs) from STA of a myelin stain of human V1 superimposed on an anisotropy‐weighted 2D orientation color map (orientation color coded between dark red = horizontal/tangential and green = vertical/radial) (B) and its corresponding myelin stain (C), with six myeloarchitectural layers labeled from superficial layer 1 (bottom) to deep layer 6 (top).^55^ (Reproduced with permission from Budde and Annese^55^ and Seehaus et al.^58^)

Validation studies on tractography results have been performed with different approaches, including the use of hardware phantoms,^50^, ^51^, ^52^ computational phantoms, *in vivo* data^53^, ^54^ and histological validation on *ex vivo* biological tissue. We focus on the latter here. Validation of the accuracy of FODs in human post‐mortem tissue was facilitated by automated 2D structure tensor analysis (STA) to detect local image orientation in histological sections.^55^, ^56^ Budde and Frank^56^ initially applied this method to the rat brain and showed that diffusion tensor imaging (DTI)‐derived FA correlates well with STA‐derived anisotropy values. This agrees with high FOD accuracy to an orientation error of approximately 5°–6° when compared with orientational histograms of manually traced axon segments in an earlier rat brain tissue study.^57^ In addition, they demonstrated the construction of FODs from STA and illustrated the effects of multiple fiber bundles and intravoxel orientation dispersion. By applying STA to human brain sections obtained from a digital public brain bank, Budde and Annese^55^ showed the complexity of human WM fibers at microscopic resolution and the effects of partial volume and small‐scale orientation dispersion on FOD estimates at lower resolutions obtainable with dMRI. Seehaus et al.^58^ applied STA to myelin‐stained human brain sections aimed at validating DTI orientation estimates in both WM and GM (Figure 4A). The agreement was strongest in WM, particularly in unidirectional fiber pathways in which high FA values were linked to high myelin density and a sharply tuned histological orientation profile. However, DTI orientations broke down in WM regions with multiple differently oriented fiber populations (Figure 4A.b). In addition to WM fiber orientation, estimates of within‐voxel fiber dispersion have been validated using optical microscopy. Diffusion‐based estimates of fiber dispersion agreed well with myelin stains, but not when a contribution from glial stains was included, suggesting that dispersion estimates predominantly reflect axons and not glial processes.^59^ In cortical GM, the agreement was good in the deeper layers, highlighting the relatively dominant radial fiber orientations even at low FA, but tensor anisotropy and orientation accuracy were lost in cortical layers with both radial and tangential intracortical fibers (Figure 4A.a). This layer‐specific organization of radial and tangential fibers was also highlighted by Budde and Annese^55^ (Figure 4B,C).

STA has recently been extended to a 3D technique, making use of confocal optically sectioned microscopy.^60^, ^61^ Although bias from anisotropic depth sampling and resolution in confocal microscopy must be carefully avoided, the resulting true 3D orientation information permits even more detailed validation of dMRI‐derived FODs, especially in the out‐of‐plane direction. These techniques have recently been leveraged to compare a broad range of diffusion‐based models, demonstrating the strengths and weaknesses of different approaches, as well as the current shortcomings of all current models (for example, robust and accurate estimation of multiple peaks in FOD).^62^ This brings the information gained from STA closer to techniques that derive 3D orientation from the optical birefringence of myelin, such as optical coherence tomography (OCT)^63^, ^64^, ^65^ and polarized light imaging (PLI).^66^ Wang et al.^65^ employed a serial optical coherence scanner (SOCS), a tissue slicer integrated with multi‐contrast OCT, and reported that fiber orientations showed good agreement between the DTI and SOCS measures; however, DTI did not capture complex (multi‐orientation) patterns as well as SOCS.

**Figure 5:**
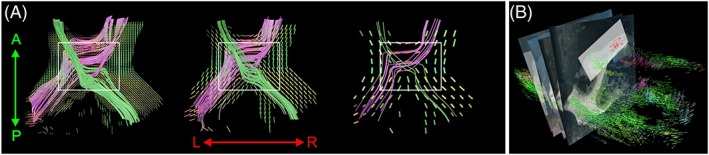
Validation of tractography. (A) The effect of resolution on diffusion tensor imaging (DTI) streamline tractography in the human optic chiasm from downsampled isotropic resolutions: 312.5 μm, left; 625 μm, middle; 1250 μm, right.^67^ (B) An illustration of two‐dimensional (2D) histological section (micrographs with green and red color indicating carbocyanine dye) alignment in the human temporal lobe to diffusion magnetic resonance imaging (dMRI) data of the same tissue for validation [primary DTI eigenvectors rendered as three‐dimensional (3D) cylinders with orientation color coding (red, left–right; green, anterior–posterior; blue, inferior–superior)].^68^ (Reproduced with permission from Roebroeck et al.^67^ and Seehaus et al.^68^)

Moving the validation from local orientations to entire tractography streamlines, Roebroeck et al.^67^ validated streamline tractography of known human optic chiasm pathways at 9.4 T and investigated the effects of spatial resolution of the diffusion data (Figure 5A). Despite the complex structure of the fiber paths through the optic chiasm, all known connections could be tracked by a DTI line propagation algorithm, although under‐represented, over‐represented and erroneous connections were tracked. Errors made by the tractography algorithm at high resolution were shown to increase at lower resolutions closer to those used *in vivo*. Seehaus et al.^68^ combined DTI at 9.4 T with carbocyanine dye tracing in the same human temporal lobe tissue block. As axons are selectively labeled along their length, robust definitions of sensitivity and specificity for DTI tractography could be given, and its accuracy could be assessed in a receiver operating characteristic (ROC) curve analysis. Validation by histological sections required careful 2D section to 3D MRI data alignment, as illustrated for this study in Figure 5B. DTI streamline tractography was shown to have a sensitivity and specificity of greater than 80% over distances of several centimeters with voxel sizes in the hundreds of microns. In these studies (Figure 5), dMRI data were acquired with high spatial resolution, but with relatively low angular resolution and *b* value, which allowed single‐fiber analysis. In post‐mortem human high angular resolution diffusion imaging (HARDI) studies (see also Section 3.2), data with high angular resolution (≥30 directions) and relatively high *b* values (>3000–4000 s/mm^2^) were acquired, which enabled multi‐fiber analysis. As an example, high tractography accuracy was also reported in a comparative study of WM projections from ventral prefrontal cortex between human and macaque brains.^69^ In this study, Jbabdi et al.^69^ combined direct tracer measurements of entire WM trajectories in macaque monkeys with probabilistic dMRI tractography on crossing fiber orientation models, allowing explicit validation, finding an overlap in monkeys between the two techniques. However, in a study using reference atlas axonal tracer results to assess the sensitivity and specificity of several tractography algorithms for long‐range projections in the macaque brain, anatomical accuracy was highest for different tractography parameters in different tracts with overall low accuracy.^70^ In another study of the macaque brain, validating tractography accuracy with anterograde tracers and myelin stains, Reveley et al.^71^ gave dense juxta‐cortical zones of tangential fibers crossed by radial cortical insertions as a possible reason for this limited accuracy.

*Ex vivo* tractography and its validation are becoming increasingly important in the context of human neurosurgery. In an *ex vivo* mesoscopic dMRI study of surgically excised hippocampi in a mesial temporal lobe epilepsy case, Modo et al.^72^ showed that individual cell layers can be discerned in dMRI contrasts, and that DTI tractography detects extra‐ and intrahippocampal connections. The results suggest that there is an aberrant connection between the dentate gyrus and the stratum moleculare of the hippocampus, supporting the hypothesis of mossy fiber sprouting in the dentate gyrus as a reverberant excitatory network in mesial temporal lobe epilepsy. In a validation study of the dentatorubrothalamic tract (DRTT), Mollink et al.^73^ compared post‐mortem dMRI tractography with 3D histological reconstruction from serial myelin‐stained sections. ROC curve analysis showed a high sensitivity and specificity in general, and high spatial accuracy of post‐mortem probabilistic tractography of the DRTT when compared with a 3D histological reconstruction, showing prospects for the study of structure–function correlations in patients with cerebellar disorders. Plantinga et al.^74^ performed an *ex vivo* tractography study of the connections between the subthalamic nucleus, substantia nigra and globus pallidus, three nuclei of the human basal ganglia, which play an important role in motor, associative and limbic processing and have relevance for deep brain stimulation (DBS) treatments. Most of the commonly established projections of the subthalamic nucleus, substantia nigra and globus pallidus were successfully reconstructed, but tracks not shown previously and a larger degree of connectivity than previously documented were observed, showing that the results should be interpreted with caution.

### 3.2 Atlasing and mapping of subcortical structures

Recent studies have demonstrated ultrahigh‐resolution 3D dMRI of subcortical brain structures on high‐field systems based on *q*‐space sampling schemes, including single‐shell HARDI^39^, ^40^ and diffusion spectrum imaging (DSI).^75^ Using pulsed‐gradient spin‐echo acquisition at 7 T, Dell'Acqua et al.^40^ demonstrated the mapping of the mesoscale connectivity of the human cerebellum with 30 diffusion‐weighting directions and 100 μm in‐plane resolution. At this resolution, the axonal connectivity of individual cerebellar folia could be reconstructed using dMRI, and compared directly with immunohistochemistry of the same tissue to investigate the cytoarchitectural basis of diffusion properties in different layers of the cerebellar cortex.^40^

**Figure 6:**
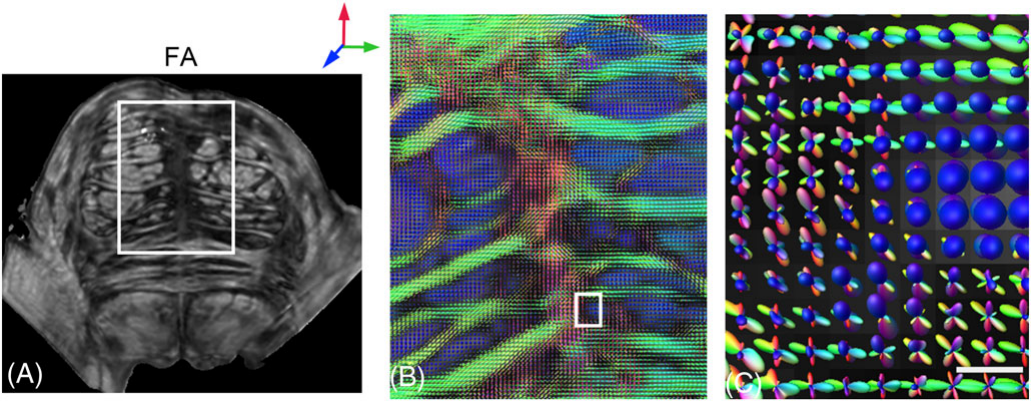
Structural organization of the human basis pontis as resolved with *ex vivo* high angular resolution diffusion imaging (HARDI) at 11.7 T.^39^ (A) Coronal slice through the fractional anisotropy (FA) map. (B) Fiber orientation distributions (FODs) reconstructed from constrained spherical deconvolution in the region corresponding to the white box in (A). Red, green and blue in color‐coded FODs indicate anterior–posterior, medial–lateral and inferior–superior orientations, respectively. The FOD map reveals interdigitating corticospinal fascicles (blue) and transverse pontine fibers (green) projecting from the pontine nuclei. (C) Zoomed‐in view of FODs in a small region (within the white box in B) shows the intravoxel crossing fiber orientations resolved with HARDI. Scale bar, 250 μm. (Data from Aggarwal et al.^39^)

Reduced partial volume effects attained with 3D isotropic resolutions also enable the reconstruction of complex intravoxel fiber orientation distributions in subcortical structures which are otherwise difficult to resolve with typical *in vivo* resolutions of approximately 1–3 mm, and are thus important for the evaluation of different diffusion processing techniques and to reliably compare diffusion modeling approaches. Wedeen et al.^75^ used DSI encoding with 515 gradient steps and 512 μm isotropic resolution to demonstrate the reconstruction of crossing fiber tracts in several subcortical structures in the *ex vivo* macaque brain at 4.7 T. Compared with the diffusion tensor formalism, DSI tractography was shown to resolve intersecting fibers in regions including the optic chiasm, cerebellar folia and brainstem in the adult macaque brain.^75^ Using 12‐shot segmented EPI at 11.7 T, Aggarwal et al.^39^ demonstrated 3D dMRI of the human brainstem with 30 diffusion‐encoding directions and 255 μm isotropic resolution. FODs obtained from single‐shell HARDI data using constrained spherical deconvolution (CSD) analysis enabled the 3D reconstruction and mapping of crossing fibers in the brainstem. Figure 6 shows complex intravoxel fiber orientation distributions resolved in the basis pontis using CSD, revealing the intricate organization of interdigitating corticospinal fascicles and transverse fibers projecting from pontine nuclei (Figure 6B,C).

In addition, high angular and spatial resolution mapping of subcortical structures lends itself to the development of advanced 3D neuroanatomical atlases as shown recently,^39^, ^76^ which can benefit the analysis and interpretation of *in vivo* dMRI datasets acquired with relatively low clinically feasible resolutions in both healthy and pathological cases. Calabrese et al.^76^ showed the feasibility of constructing a high‐resolution dMRI template of the post‐mortem human brainstem and thalamus on a preclinical 7‐T system, which was used to register *in vivo* clinical datasets from patients for electrode targeting in DBS. With recent advances in human MRI scanners equipped with stronger magnetic field gradients, e.g. the 300‐mT/m connectome scanner, *q*‐ball imaging (QBI) of the intact *ex vivo* human brain at submillimeter (0.6 mm) isotropic resolution was demonstrated in a study by McNab et al.,^38^ who showed intravoxel crossing fibers in the brainstem with orientation distribution functions obtained from *q*‐ball reconstruction.

### 3.3 Detection of GM lamination

In recent years, there has been growing interest in high‐resolution dMRI of the cortex to investigate its laminar microstructure. The defining feature of the cortex is its layered structure which shows heterogeneity across functionally distinct areas. Early *ex vivo* dMRI studies had already noted the dominant radial anisotropy over the entire depth of the primate cortex, especially early in development.^77^, ^78^ Challenges to the mapping of cortical microstructure and lamination using dMRI include sufficiently high contrast (both in terms of *b* value and angular coverage) to resolve the intrinsically low anisotropy in GM, and intravoxel partial volume effects as a result of the thickness (~1–4.5 mm) of the cortical ribbon in the human brain which is comparable with typical *in vivo* dMRI resolutions. Moreover, the underlying microstructural correlates of diffusion anisotropy in cortical GM are not clearly understood at present.

**Figure 7:**
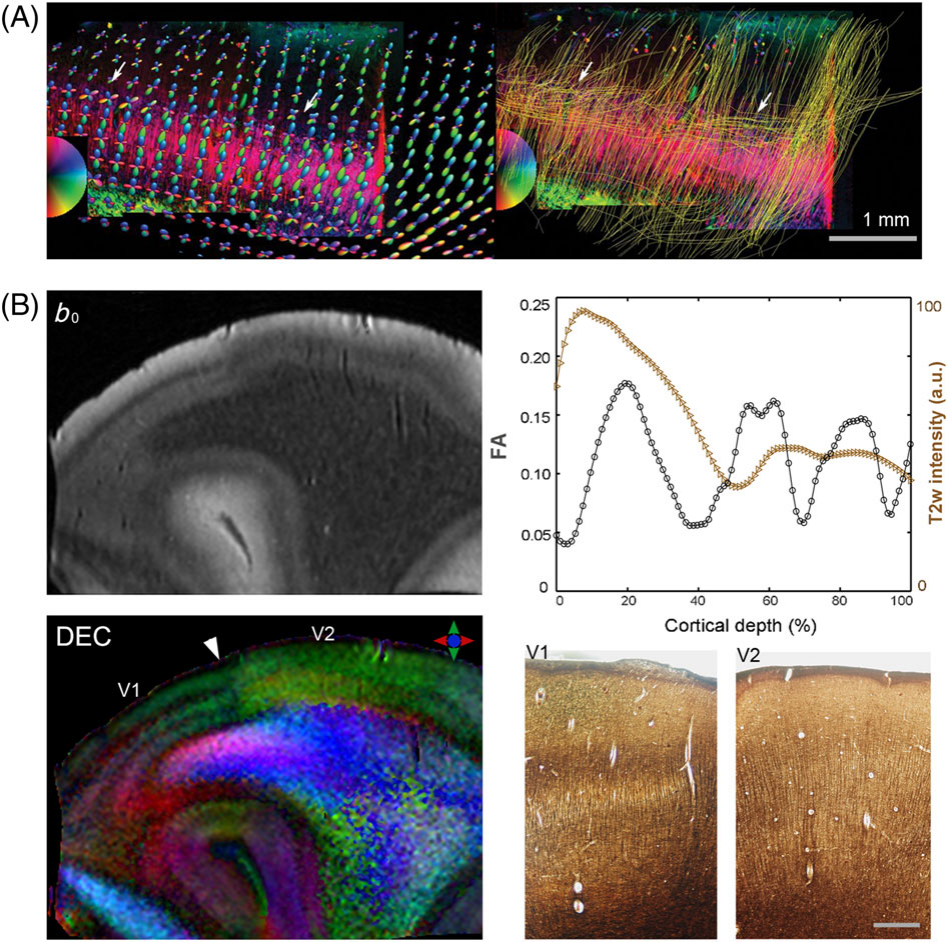
Laminar structure of the human visual cortex revealed with *ex vivo* high angular resolution diffusion imaging (HARDI). (A) A polarized light imaging (PLI) section of the primary visual area overlaid with diffusion magnetic resonance imaging (dMRI)‐derived fiber orientation distributions (FODs) (left) and intracortical fiber tracts from streamline tracking (right). Arrows indicate tangential fibers in the stria of Gennari. (B) Delineation of cortical layers and the marked transition between primary (V1) and secondary (V2) visual areas seen with dMRI at 11.7 T. Distinct intracortical layers corresponding to the stria of Gennari and inner band of Baillarger in V1 are evident in the direction‐encoded color (DEC) map, which shows excellent agreement with the cortical myeloarchitecture seen in silver‐stained sections adjacent to the V1–V2 boundary in the same specimen. The fractional anisotropy (FA) profile across the cortical depth is shown at the top right. T2w, *T*_2_‐weighted. Scale bar, 0.5 mm. (Reproduced with permission from Aggarwal et al.^45^ and Leuze et al.^81^)

Regional variation has been shown in the orientation of the primary eigenvectors of diffusion tensors in the primary sensory *versus* motor cortices in the human brain.^79^ A study by Dyrby et al.^23^ reported the delineation of parallel rims in GM of the cerebral cortex and hippocampus in the *ex vivo* porcine brain, based on multi‐fiber reconstruction with persistent angular structure (PAS) MRI. More recently, high‐field HARDI studies have demonstrated the potential of dMRI to resolve layer‐specific properties in human cortical tissue.^45^, ^80^, ^81^ In a study at 11.7 T, Kleinnijenhuis et al.^80^ showed layer‐specific variation in FA across the cortical depth and FODs with multiple components in the stria of Gennari and deeper layers of the primary visual cortex. Leuze et al.^81^ demonstrated high angular and spatial (242 μm isotropic) resolution dMRI of the human visual cortex at 9.4 T. Based on reconstructed fiber orientation distribution functions, the primary visual cortex could be differentiated into four cortical zones. These authors traced tangential intracortical fibers in the stria of Gennari, and compared the results with PLI and myelin staining, finding them in good agreement (Figure 7A). The potential of dMRI to resolve the laminar structure of the visual cortex and to probe microstructural heterogeneity across Brodmann areas was further demonstrated in a recent study by Aggarwal et al.^45^ With 3D‐GRASE acquisition at 11.7 T, this study demonstrated single‐shell HARDI at 92 μm resolution which revealed region‐specific FA profiles for the primary motor, somatosensory and visual cortices. FODs reconstructed from CSD analysis could resolve crossing radial and tangential fibers in the intracortical bands of Baillarger, which exhibited close agreement with region‐specific myeloarchitecture as seen by comparison with silver impregnation of the same cortical areas. Figure 7 shows an example of the laminar architecture of the human visual cortex resolved with high angular and spatial resolution dMRI, and the clear demarcation between primary and secondary visual areas based on their diffusion characteristics (Figure 7B).

These recent studies provide accumulating evidence that layer‐specific diffusion properties and FODs reconstructed using HARDI can be used to discriminate between different cortical areas in the human brain, e.g. the primary somatosensory and motor cortices, and primary *versus* secondary visual areas. Deciphering the layer‐specific diffusion properties in cortical GM has important potential applications for 3D mapping of cortical myeloarchitecture in the human brain. An early application of this was reported by Bastiani et al.,^24^ who showed automatic segmentation of layer complexes and myeloarchitectonic areas in human cortex from *ex vivo* dMRI. Further, an understanding of the microstructural basis of the diffusion nuclear magnetic resonance (NMR) signal in cortical GM is also important for the meaningful interpretation of *in vivo* dMRI contrasts in the human cortex with increasing resolution,^82^ as high performance gradients and more sensitive RF coils become available.

## 4 CONCLUSION AND OUTLOOK

The sophistication of specific tissue preparation techniques and pulse sequences has increased to a level at which *ex vivo* dMRI has quickly become an important tool in brain anatomy studies with mesoscopic spatial resolution. Whole *ex vivo* human brain dMRI has already been reported at 400 μm isotropic resolution,^37^ which is a factor of 18 higher 3D resolution than the 7T Human Connectome Project (HCP) *in vivo* dMRI datasets. With continuing advances in both *in vivo* and *ex vivo* techniques, *ex vivo* dMRI is likely to continue leading the way for *in vivo* acquisitions in terms of resolution. It is important to realize that high‐quality *ex vivo* dMRI data do not come easily. Achievable quality depends on the quality and preparation of the sample (its PMI and mode of fixation), pulse sequences tailored to *ex vivo* imaging, long acquisitions (often days) and sometimes bespoke RF coils^83^ or other hardware. This also has the consequence that sample numbers in *ex vivo* dMRI are often low, something more often seen in classical anatomy studies, which is challenging for the generalization of the results. In addition, combining high spatial resolution, high angular resolution and high *b* values is challenging, as in *in vivo* imaging, which is nonetheless necessary for modern (crossing) fiber orientation analysis and tractography steps.

*Ex vivo* dMRI of the human brain has had an important role in the validation of the accuracy and precision of both fiber orientation estimates and tractography results. Both of these areas are likely to expand and be combined with more sophisticated histological techniques. Technical advances in histology and microscopy are increasing both the level of detail and the field of view that can be investigated. Recent efforts have already shown the potential of classical cytoarchitectonic staining of an entire brain at the very high resolution of 20 μm,^84^ with more individual datasets currently underway. Combining this extensive microscopy assay with whole‐brain dMRI before sectioning could provide powerful multi‐modal datasets. Furthermore, optical tissue clearing techniques have enabled the investigation of cell structure and connectivity in large intact tissue samples with light microscopy techniques.^85^, ^86^, ^87^, ^88^ Interestingly, these same clearing techniques have provided insights into the source of the diffusion signal by revealing that the removal of lipids whilst preserving proteins largely removes diffusion‐based contrast.^89^ The validation of dMRI fiber orientation and tractography results stands to gain considerably by combination with techniques such as PLI.^66^ Here, it should be noted that both techniques, although at different intrinsic resolutions, have the same basic tractography problem to solve, starting from local fiber orientations. Another technique which can help in the validation of dMRI‐based tractography results is OCT.^90^ As an advantage over PLI, OCT does not require the tissue to be sectioned before acquisition, and it is therefore less prone to deformation artifacts. In addition, OCT can provide both cytoarchitectural and myeloarchitectonic information.^64^

*Ex vivo* dMRI validation studies using human tissue have largely focused on fiber orientation estimates and tractography, as reviewed above. However, it is imperative to note that dMRI is an exquisite technique not only to probe orientational anisotropy at the macro‐ and mesoscales at the level of imaging voxels, but also to probe brain microstructure (see other contributions to this issue). The fitting of biophysical multi‐compartment models to the dMRI signal provides estimates of microstructural parameter maps, chief amongst which are axonal density maps (related to intra‐axonal volume fractions of water pools showing restricted diffusion) and axonal diameter distribution (ADD) maps. It should be noted that the inference of microscale connectivity features, such as axon densities and diameters (cf. Figure 1), is achieved through indirect modeling of the signal over multiple measurements and inferring statistics of these features over macroscale or mesoscale voxels. These inferred microscale parameters are therefore best validated by light microscopy or, most commonly, electron microscopy methods, which directly resolve these features at the level of individual axons by the acquired spatial resolution. Many studies introducing diffusion microstructure models have combined *ex vivo* dMRI with one of these two techniques, almost exclusively in animals.^91^, ^92^ Continued validation of diffusion microstructure models could benefit from studies of human tissue, as this would validate a microstructural environment relevant to *in vivo* human applications. This would place considerable requirements on the *ex vivo* dMRI acquisitions, as microstructural models tend to require a larger number of distinct *b* values and, in the case of axon diameter indices, multiple diffusion times.

Human *ex vivo* dMRI studies are likely to continue to expand to larger tissue samples and the intact human brain in larger bore‐size systems, such as human UHF systems, particularly with technological advances in RF coils custom built for post‐mortem tissue, gradient hardware and higher magnetic field strengths. This would provide the invaluable context of the entire human brain with both its long‐range connections and cortical fields of view extending over many square centimeters of GM. However, the highest quality *ex vivo* acquisitions are only possible on very well‐preserved human brain specimens, which require short PMIs. In addition, such large fields of view and simultaneously high‐resolution acquisitions will also require the establishment of very high SNR efficiency acquisition schemes. Furthermore, the concomitant increase in imaging matrix size is likely to tax image reconstruction and data processing and analysis routines, pushing them into the big data regime. Ultimately, with these and other described advances in the field, *ex vivo* dMRI is likely to be pivotal in bridging the gap in knowledge that exists between *in vivo* estimates of macroscopic connectivity and underlying human brain tissue microstructure.

 

 
